# Healthcare use and healthcare costs for patients with advanced cancer; the international ACTION cluster-randomised trial on advance care planning

**DOI:** 10.1177/02692163221142950

**Published:** 2022-12-14

**Authors:** Ida J Korfage, Suzanne Polinder, Nancy Preston, Johannes JM van Delden, Sandra (A)JLM Geraerds, Lesley Dunleavy, Kristof Faes, Guido Miccinesi, Giulia Carreras, Caroline Moeller Arnfeldt, Marijke C Kars, Giuseppe Lippi, Urska Lunder, Ceu Mateus, Kristian Pollock, Luc Deliens, Mogens Groenvold, Agnes van der Heide, Judith AC Rietjens

**Affiliations:** 1 Department of Public Health, Erasmus MC, University Medical Center Rotterdam, Rotterdam, The Netherlands; 2 International Observatory on End of Life Care, Division of Health Research, Lancaster University, Lancaster, UK; 3 Julius Centre for Health Sciences and Primary Care, UMC Utrecht, Utrecht, The Netherlands; 4 End-of-Life Care Research Group, Vrije Universiteit Brussel (VUB) & Ghent University, Brussels, Belgium; 5 Clinical Epidemiology, Oncological network, prevention and research Institute (ISPRO), Florence, Italy; 6 Department of Public Health, University of Copenhagen, Copenhagen, Denmark; 7 Department of Palliative Medicine, The Research Unit, Bispebjerg Hospital, Copenhagen, Denmark; 8 Tuscany Region Health Agency, Italy; 9 University Clinic of Respiratory and Allergic Diseases Golnik, Golnik, Slovenia; 10 Division of Health Research, Lancaster University, Lancaster, UK; 11 School of Health Sciences, University of Nottingham, Nottingham, UK

**Keywords:** Advance care planning, health care costs, cancer, delivery of health care, randomised controlled trial

## Abstract

**Background:**

Advance care planning supports patients to reflect on and discuss preferences for future treatment and care. Studies of the impact of advance care planning on healthcare use and healthcare costs are scarce.

**Aim:**

To determine the impact on healthcare use and costs of an advance care planning intervention across six European countries.

**Design:**

Cluster-randomised trial, registered as ISRCTN63110516, of advance care planning conversations supported by certified facilitators.

**Setting/participants:**

Patients with advanced lung or colorectal cancer from 23 hospitals in Belgium, Denmark, Italy, the Netherlands, Slovenia and the UK. Data on healthcare use were collected from hospital medical files during 12 months after inclusion.

**Results:**

Patients with a good performance status were underrepresented in the intervention group (*p*< 0.001). Intervention and control patients spent on average 9 versus 8 days in hospital (*p* = 0.07) and the average number of X-rays was 1.9 in both groups. Fewer intervention than control patients received systemic cancer treatment; 79% versus 89%, respectively (*p*< 0.001). Total average costs of hospital care during 12 months follow-up were €32,700 for intervention versus €40,700 for control patients (*p* = 0.04 with bootstrap analyses). Multivariable multilevel models showed that lower average costs of care in the intervention group related to differences between study groups in country, religion and WHO-status. No effect of the intervention on differences in costs between study groups was observed (*p* = 0.3).

**Conclusions:**

Lower care costs as observed in the intervention group were mainly related to patients’ characteristics. A definite impact of the intervention itself could not be established.

**What is already known about the topic?**

- Advance care planning has been found to be associated with reduced healthcare use and costs.
- Two studies have addressed this in patients with cancer; one found an association with reduced healthcare use and costs, the other found no effect.

**What this paper adds**

- Average hospital care costs per patient at 12 months after inclusion were €32,700 in the advance care planning intervention group compared to €40,700 in the control group.
- Lower healthcare use and costs in the intervention group were significantly associated with patient characteristics such as country of residence and worse performance status, and not with the advance care planning intervention.
- Healthcare use and cost patterns differed per country.

**Implications for practice, theory or policy**

- A definite impact of the intervention itself could not be established.
- The association of patient characteristics with healthcare use and healthcare costs was more outspoken than their association with the intervention.

## Introduction

According to the definition of the WHO, palliative care should be tailored to patients’ individual preferences, addressing their goals and needs concerning care, symptom control, psychosocial support, spiritual support, and practical issues.^1^ Advance care planning might be supportive in the process of making decisions about future medical care in case of serious incurable illness. Advance care planning is defined as a process that enables individuals to define goals and preferences for future medical treatment and care, to discuss these goals and preferences with family and health-care providers, and to record and review these preferences if appropriate.^2^ An important aim of advance care planning is to better align care to patients’ preferences, since care for patients in the final stages of life is not always consistent with their treatment goals.^3^

A review focusing on advance care planning with people with advanced cancer identified two studies on advance care planning and costs.^4^ One of these, a randomised clinical trial among 213 patients with stage 3 or 4 or recurrent cancer in the United States, found an association of assisting patients in establishing preferences for end-of-life care and reduced healthcare use and costs.^5^ The other study focused on advanced directives of 336 patients with advanced cancer, also in the United States, and found no association between completion of documents and costs.^6^ The review concluded that advance care planning and goals-of-care conversations were associated with less ‘aggressive’ (i.e. intensive, life-prolonging) and less costly end-of-life care.

Much is thus still unknown about the association of advance care planning with the use and costs of care among patients with advanced cancer. Studies on advance care planning and healthcare use and costs among other patient populations^5–32^ showed that advance care planning tends to be associated with reduced healthcare costs.^5–9,11–13,15,17,18,21,23–25,28–30^ Cost savings were related to people choosing less invasive medical interventions after having been engaged in advance care planning^33^ or to people being less often hospitalised or for shorter periods.^25,29,30^ However, advance care planning can also lead to increased care use and costs as shown in a modelling study among patients with renal diseases.^26^ In a study of healthcare claims and advance care planning, mixed effects were observed; higher rates were found of admission to hospices and hospital in those engaging in advance care planning, but lower rates of chemotherapy.^34^

We aimed at filling the international knowledge gap regarding the effect of advance care planning on use of care of patients with advanced cancer. To prevent contamination we applied cluster-randomisation, with 23 hospitals as clusters. In six European countries, we evaluated the ACTION Respecting Choices (RC) advance care planning intervention among adult patients with advanced lung or colorectal cancer.^35^ No significant effects of advance care planning on the primary outcomes of quality of life and symptoms were found.^35^ In this paper, we present a detailed analysis of how this comprehensive advance care planning programme impacted healthcare use and associated costs.

## Methods

### Setting

**Figure 1.**
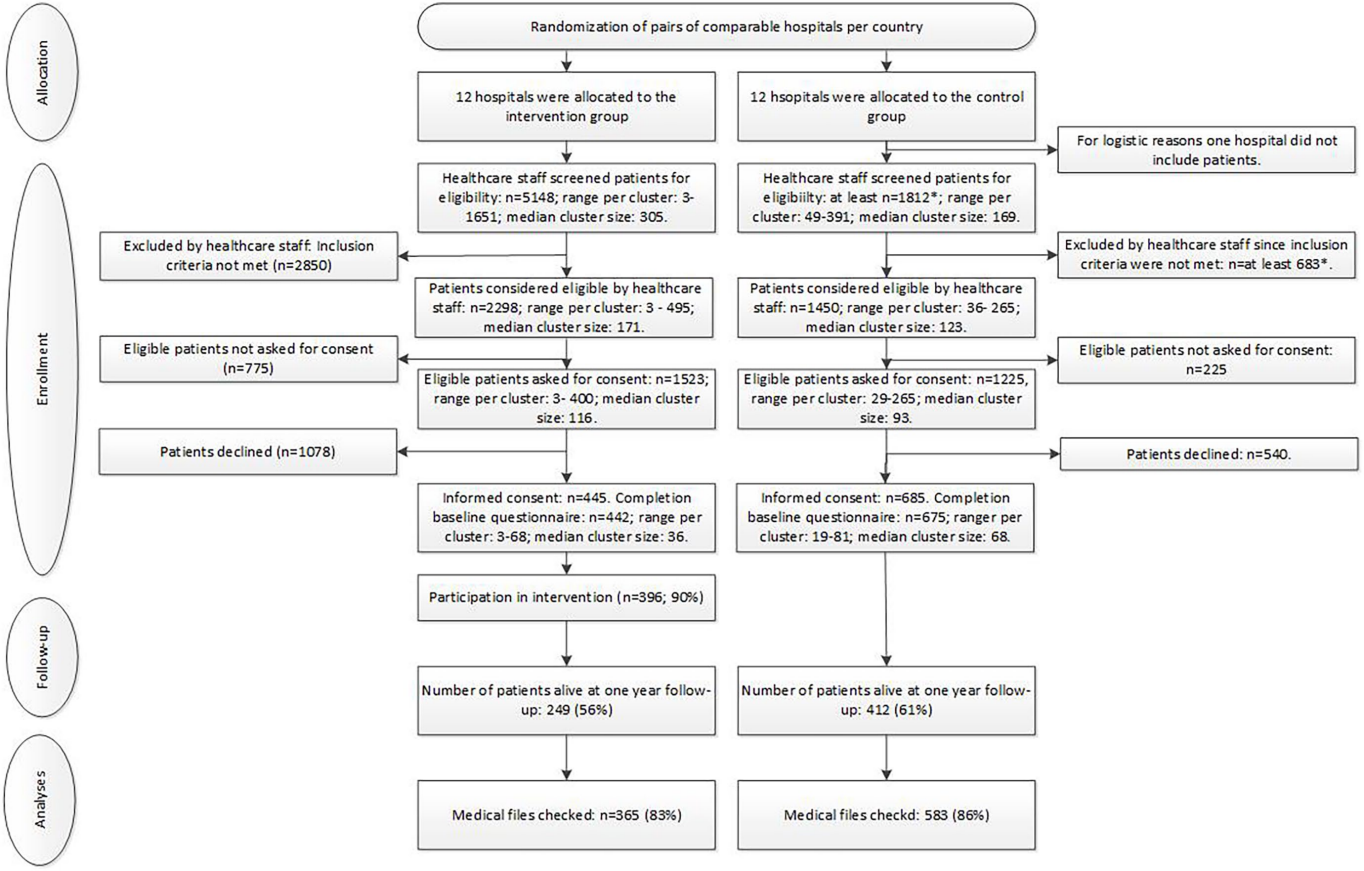
Consort flowchart.

The ACTION trial was a multicentre cluster-randomised controlled trial in 23 hospitals in six European countries (Belgium, Denmark, Italy, the Netherlands, Slovenia and the United Kingdom, see Figure 1).

We selected comparable hospitals and per country we conducted pairwise randomisation of, for example, academic hospitals and of non-academic hospitals.

### Participants and advance care planning programme

Adult, competent patients with advanced lung (stage III or IV) or colorectal cancer (stage IV) and an estimated life expectancy of at least 3 months were eligible for participation (see Supplemental Appendix 1 for the inclusion criteria). Members of the patient’s usual care team assessed the eligibility of patients, and eligible patients were asked to consider participation in the ACTION trial. Patients in control hospitals were informed that ACTION focused on preparing patients for decision-making about care, and that they would receive usual care. Those in the intervention hospitals received information about the intervention. The ACTION RC advance care planning intervention^36^ was an adapted and integrated version of the RC First Steps and Advanced Steps facilitated advance care planning conversations, which are part of the more comprehensive RC advance care planning programme that was developed in La Crosse, Wisconsin, in the USA (https://respectingchoices.org/).

The ACTION RC advance care planning intervention included three components:

1. Facilitated advance care planning conversations using structured guides

Certified facilitators used scripted conversation guides to support patients and their relatives (personal representatives) in exploring their understanding of the illness, reflecting on their goals, values and beliefs, and discussing their preferences for future treatment and care. The intervention could involve one or two conversations, with or without a personal representative.

The facilitators measured the duration of the advance care planning conversations they conducted.

2. My Preferences form

The My Preferences form (Supplemental Appendix 2) was a study specific form to document preferences. Depending on local regulations, the My Preferences form could be considered as a formal AD. It aligned with the topics in the advance care planning conversation guides and consisted of open sections addressing ‘Living well’, ‘Worries and fears’, ‘Beliefs’ and ‘Hopes’, and structured sections to indicate preferences regarding cardio-pulmonary resuscitation (CPR), goals of future care, and final place of care. Patients were offered the option of completing a My Preferences form, either during or after the advance care planning conversation.

3. Information leaflets

Leaflets about advance care planning and the role of the personal representative were provided to intervention participants. Where relevant, patients also received leaflets about CPR, artificial ventilation or artificial feeding.

Data were collected between 2015 and 2018. More details of the study design, methods and main findings have been reported previously.^35,36^

### Economic evaluation

No significant effect of advance care planning on the primary effect outcome measures (quality of life and symptoms) was found.^35^ Since we found that ACP conversations did not have an impact on patients’ quality of life, coping or involvement in decision-making processes, we did not perform a cost-effectiveness study, but a cost-minimisation analysis (CMA). CMA helps to find the treatment with the lowest cost, which then will be the treatment of choice.

We investigated the difference in use of hospital care and associated costs between the study groups from a hospital perspective. To analyse the costs of healthcare use during 12 months after inclusion, we collected detailed information on hospital care consumption, including emergency department (ED) visits, hospital stays, ICU care; diagnostic procedures (e.g. blood transfusion or CT scan), medical interventions (e.g. surgery or CPR), and medication. Data were collected from participants’ hospital files using a standardised checklist. This checklist (Supplemental Appendix 3) was pilot-tested to verify whether relevant care items were accessible in medical files in all six countries and to reduce inter-rater differences in interpretations between researchers who collected these data. The checklist was completed for 12 months following study inclusion, until patient’s death (if the patient died within that period), or until the end of data-collection.

A manual was developed in the project, in which we described in detail which unit prices for hospital care, diagnostic procedures, and medical interventions should be collected. The preferred perspective was the hospital perspective. The preferred source were national guidelines, providing reference prices as a proxy for real costs, followed by pricelists of hospitals. A reference price is an average unit price as estimated on the basis of large, diverse populations that can be directly used to value resource quantities. All countries provided unit prices based on this manual, as far as possible.

Direct costs of medical care were calculated by multiplying their quantity with the corresponding unit prices per country. If unit prices were unavailable for a country, the price was calculated as the mean of the available reference prices for other countries. The mean was then corrected for the purchasing power parities (PPP) for general domestic product (GDP) per country. Costs were adjusted for inflation and reported in 2018 euros. Finally, unit prices per country were used to calculate overall costs.

### Statistical analysis

Statistical analyses were conducted according to the intention-to-treat principle. Only data of patients whose hospital files had been checked were included in the data analyses. Personal characteristics were compared at baseline between study groups using chi-square tests for categorical variables and Mann-Whitney *U* tests for continuous variables. Costs of medical care were compared between study groups and subgroups based on country using independent sample *T*-test with bootstrapping, drawing 1000 samples. The bootstrapping was used as cost data is typically skewed and unlikely to meet the normality assumption underlying the *t*-test. Differences were considered significant if *p*< 0.05. We generated log-linked gamma generalised linear mixed models (GLMM) to investigate association between variables and costs. These models included a random intercept for hospital, to adjust for clustering of patients within hospitals. Patients for whom complete medical file data was available, that is, for the entire period of 12 months following study inclusion or until their death (if they died within 12 months after inclusion) were included in this complete-case analysis. Next, a multivariable GLMM was generated including all variables that had been considered in the univariable models. In addition, as sensitivity analysis, we investigated the association between variables and costs again, excluding patients who died during the 12-month follow-up period. Analyses were performed using IBM SPSS statistics V.23 and R V.3.2.3.

### Ethics

Ethical approval for the study was obtained from the Research Ethics Committee (REC) of the coordinating centre (Erasmus MC), as well as RECs in all participating countries. The trial was registered in the International Standard Randomised Controlled Trial Number registry (ISRCTN63110516) per 10/3/2014. A Data Safety Monitoring Board conducted four interim analyses.

## Results

### Procedures

Between 2015 and 2018, 3748 patients were considered eligible, 2748 (73%) were asked to participate, and 1135 of these 2748 (41%) provided consent to participate in our study. Of these, five withdrew their consent. The recruitment rate was 29% in the intervention group (445/1523) and 56% in the control group (685/1225). Thirteen patients who were included did not complete any questionnaires.

Hospital file analyses were conducted for 365/442 (83%) patients in the intervention group and 583/675 (86%) patients in the control group. Data of these 948 patients were included in the analyses, see Figure 1. For 351 intervention group patients and 572 control group patients, complete medical file data was available and data of these 923 patients were included in the complete-case analyses. Files of 169 participants could not be accessed for reasons unrelated to their condition: either files could not be checked due to end of data-collection (*n* = 62 in the intervention group, *n* = 77 in the control group) or files could not be checked for logistical reasons (*n* = 15 in the intervention group, *n* = 15 in the control group).

First (*n* = 396) and second (*n* = 116) ACP conversations as conducted with patients in the intervention group lasted on average 1 h and 12 min. Third conversations (*n* = 2) lasted 30 min on average. Depending on the country, ACP conversations were conducted by psychologists, doctors or nurses. The costs of conducting ACP conversations therefore range from € 16 to € 122 per hour (Box 1).

### Characteristics

Table 1 presents characteristics of 948 participants of whom data concerning use of medical care were available. Their mean age was 66.1 years (SD: 9.9; *p* = 0.81), 378 (40%) were female (*p* = 0.94). Sociodemographic characteristics were comparable between study groups, except for country of residence (*p*< 0.001). At time of inclusion, some clinical characteristics differed between groups, for example, fewer intervention patients received systemic treatment (79%) compared to control patients (89%; *p*< 0.001), and fewer intervention patients had WHO performance status 0 (25%) than control patients (40%; *p*< 0.001), see Table 1.

**Table 1.** Sociodemographic and clinical characteristics of ACTION participants whose hospital files were available (*n* = 948).

	Intervention group (*n* = 365)	Control group (*n* = 583)	*p-*Value*
Sociodemographic characteristics			
Age (years), mean (SD)	66.0 (10.37)	66.2 (9.56)	0.81
Range	[18, 89]	[30, 91]	
*Missing*	*0*	*3*	
Years of education, mean (SD)	13.3 (4.49)	13.2 (4.59)	0.75
*Missing*	*48*	*84*	
Female gender, *n* (%)	145 (39.7)	233 (40.0)	0.94
Living with a spouse, *n* (%)	249 (68.2)	431 (73.9)	0.05
*Missing*	*9*	*14*	
Having children, *n* (%)	311 (85.2)	500 (85.8)	0.92
*Missing*	*7*	*6*	
Religion, *n* (%)			0.17
Religious	169 (46.3)	286 (49.1)	
Not religious	147 (40.3)	207 (35.5)	
Prefers not to specify	39 (10.7)	81 (13.9)	
*Missing*	*10*	*9*	
Considering oneself member of minority group, *n* (%)	2 (0.5)	7 (1.2)	0.50
* Missing*	*16*	*18*	
Country of residence, *n* (%)			<0.001*
Belgium	60 (16.4)	114 (19.6)	
Denmark	65 (17.8)	66 (11.3)	
Italy	30 (8.2)	100 (17.2)	
The Netherlands	61 (16.7)	148 (25.4)	
Slovenia	60 (16.4)	20 (3.4)	
The United Kingdom	89 (24.4)	135 (23.2)	
Clinical Characteristics			
Diagnosis, *n* (%)			<0.001*
Small cell lung cancer, stage III or IV	60 (16.4)	40 (6.9)	
Non-small cell lung cancer, stage III or IV	173 (47.4)	251 (43.1)	
Colon cancer, stage IV	103 (28.2)	214 (36.7)	
Rectal cancer, stage IV	29 (7.9)	78 (13.4)	
*Missing*	*1*	*2*	
Years since diagnosis, mean (SD)	1.2 (1.7)	1.6 (2.0)	<0.001*
* Missing*	*4*	*3*	
Years since diagnosis of current stage, mean (SD)	0.6 (0.9)	1.0 (1.4)	<0.001*
* Missing*	*8*	*13*	
Receiving systemic treatment,^a^*n* (%)	289 (79.2)	518 (88.9)	<0.001*
* Missing*	*1*	*1*	
WHO performance status,^b^*n* (%)			<0.001*
0 Fully active	91 (24.9)	232 (39.8)	
1 No heavy physical work	199 (54.5)	286 (49.1)	
2 Up for more than half of the day	62 (17.0)	50 (8.6)	
3 In bed/sitting more than half of the day	7 (1.9)	7 (1.2)	
* Missing*	*6*	*8*	
Survival			
Died during 12-month follow-up, *n* (%)	162 (44)	233 (40)	0.010
Average time between inclusion and death, months (SD)	5.8 (2.9)	5.7 (3.2)	

SD: standard deviation.

* Significant at 5% level.

a Includes chemotherapy, immunotherapy, and targeted therapy.

b Score 0: Fully active, more/less as before illness; Score 3: In bed/sitting in a chair for more than half the day and needs some help in looking after him/herself.

During the 12-month follow-up period 162 of 365 patients in the intervention group died (44%) and 233/583 (40%) in the control group, *p* = 0.010. Average time between study inclusion and death was 5.8 months in the intervention group versus 5.7 in the control group, *p* = 0.09.

### Healthcare use and costs

Table 2 gives an overview of the use and costs of hospital care per participant. These results are impacted by differences in hospital costing systems. We refer to Supplemental Appendix 1 for unit prices per country and to Table 3 for a comparison of use of care and average costs between countries. The total mean costs of medical care during 12 months of follow-up were lower in the intervention than in the control group (€32,724 vs €40,741 respectively, *p* = 0.037 with bootstrap analyses).

**Table 2.** Average use and costs of medical care per participant during 12 months of follow-up (*n* = 948).

	Intervention group (*n* = 365)			Control group (*n* = 583)	
Cost by category	Unit price [Min, Max] (€)^a^	Average quantity	Average total costs (€)	Average quantity	Average total costs (€)
		Mean [IQR]		Mean [IQR]	
Hospitalisation					
Hospital ward stay	[331, 768]	9.0 [0, 14]	5074	7.8 [0, 11]	4601
ICU care	[1071, 1418]	0.1 [0, 0]	90	0.1 [0, 0]	113
Total hospital stay		9.0 [0, 14]	**5052**	7.8 [0, 11]	**4578**
Diagnostic procedures					
Ultrasound	[40, 88]	0.5 [0, 1]	36	0.4 [0, 1]	29
X-ray	[14, 67]	1.9 [0, 3]	75	1.9 [0, 3]	78
MRI scan	[177, 341]	0.6 [0, 1]	137	0.3 [0, 0]	65
PET scan	[176, 945]	0.2 [0, 0]	107	0.2 [0, 0]	96
CT scan	[136, 212]	3.3 [1, 4]	475	3.6 [2, 5]	502
Bone scan	[139, 228]	0.1 [0, 0]	12	0.1 [0, 0]	12
Venepuncture for lab	[2, 186]	15.7 [4, 22]	1073	17.6 [7, 24]	1242
Endoscopy	[40, 594]	0.1 [0, 0]	29	0.1 [0, 0]	27
Bronchoscopy	[107, 2202]	0.0 [0, 0]	4	0.0 [0, 0]	8
Biopsy	[50, 322]	0.1 [0, 0]	13	0.1 [0, 0]	19
Total diagnostics			**1958**		**2076**
Clinical interventions^b^					
Surgery (no.)		0.3 [0, 0]		0.2 [0, 0]	
Intravenous chemotherapy (days)	[1010, 1618]	6.6 [0, 10]	9416	7.2 [0, 10]	10,181
Oral chemotherapy (days)	[196, 380]	12.7 [0, 0]	3701	11.9 [0, 0]	3049
Radiation therapy (days)	[50, 4266]	2.0 [0, 0]	5482	1.2 [0, 30]	2580
Immunotherapy (days)	[2515, 4029]	1.5 [0, 0]	5248	3.1 [0, 1]	11,088
Targeted therapy (days)	[70, 2276]	2.7 [0, 0]	1850	6.3 [0, 0]	7108
Cardiopulmonary resuscitation^c^	[195, 313]	0.0 [0, 0]	6	0.0 [0, 0]	2
Artificial nutrition (days)^d^	[46, 455]	0.8 [0, 0]	191	0.9 [0, 0]	227
Artificial hydration (days)^e^	[8, 13]	2.2 [0, 1]	25	1.5 [0, 0]	17
Specialist palliative care; *n* (%)		134 (36.7%)		160 (27.4%)	
Total medical interventions			**25,619**		**34,001**
Medication^f^					
Antibiotics	[7, 16]	7.0 [0, 7]	110	6.2 [0, 7]	99
Total costs hospital care			**32,724**		**40,741**

a [Minimum price, maximum price] of cost prices from Belgium, Denmark, Italy, the Netherlands, Slovenia, and the UK.

b Radiotherapy and artificial ventilation were not applicable.

c Information missing: intervention group *n* = 34; control group *n* = 30.

d Information missing: intervention group *n* = 36; control group *n* = 40.

e Information missing: intervention group *n* = 44; control group *n* = 73.

f Only costs of antibiotics. Number of participants for whom this information is missing: *n* = 127 (intervention group *n* = 50, control group *n* = 77).

Bold entries indicate total amounts per section and overall total amount.

**Table 3.** Comparison of use of care and average costs^#^ (€) [and interquartile range (IQR)] between study groups and between countries.

Healthcare use and costs [IQR]	Belgium		Denmark		Italy		The Netherlands		Slovenia		United Kingdom	
	ACP (*n* = 60)	Control (*n* = 114)	ACP (*n* = 65)	Control (*n* = 66)	ACP (*n* = 30)	Control (*n* = 100)	ACP (*n* = 61)	Control (*n* = 148)	ACP (*n* = 60)	Control (*n* = 20)	ACP (*n* = 89)	Control (*n* = 135)
Hospital stay - Average number of days (IQR) - Average costs per patient - IQR of average costs	in EACH cell of this table the content needs to be placed one row LOWER 15.3 [1, 23] 7845 328, 12,949	10.9 [0, 16] 5925 0, 7691	10.5 [1, 16] 7951 384, 12,106	9.1 [0, 13] 6990 0, 9595	7.9 [0, 10] 4415 0, 5365	4.7 [0, 7] 2528 0, 2801	6.6 [0, 9] 3971 0, 5976	7.1 [0, 10] 3619 0, 4942	7.4 [0, 13] 3378 0, 5901	6.6 [0, 12] 2182 0, 3885	6.7 [0, 10] 3137 0, 4289	7.9 [0, 14] 5186 0, 9310]
Chemotherapy (oral + IV) – Average number of days (IQR) - Average costs per patient - IQR of averagecosts	40.3 [2, 28] 19,685 2765, 23,499	29.3 [2, 35] 21,809 2765, 38,358	23.3 [6, 23] 24,110 6930, 34,794	21.1 [3, 32] 25,951 4855, 42,077	27.1 [8, 25] 19,444 9171, 29,651	9.4 [0, 12] 8107 0, 13,359	10.5 [0, 9] 6828 0, 11,900	16.7 [0, 9] 7977 0, 11,200	1.8 [0, 3] 1768 0, 3030	10.6 [0, 1] 2856 0, 758	16.7 [0, 12] 9797 0, 9309	19.3 [0, 15] 10,232 0, 14,021
Number of people receiving specialist palliative care (%) ^a^	24 (40.0)	43 (37.7)	26 (40.0)	21 (31.8)	5 (16.7)	18 (18.0)	10 (16.4)	7 (4.7)	25 (41.7)	14 (70.0)	44 (49.4)	57 (42.2)
Targeted Therapy - Average number of days (IQR) -Average costs per patient - IQR of average costs	0 0 n.a.	3.6 [0, 0] 3433 0, 0	5.4 [0, 0] 6042 0, 0	1.6 [0, 0] 1727 0, 0	3.0 [0, 0] 6752 0, 0	8.4 [0, 0] 19,013 0, 0	2.8 [0, 0] 831 0, 0	5.4 [0, 0] 1587 0, 0	6.1 [0, 0] 423 0, 0	0 0 n.a.	[0, 0] 11 0, 0	11.5 [0, 0] 11,095 0, 0
CT-scan - Average number of scans (IQR) - Average costs per patient - IQR of average costs	4.3 [2, 6] 592 279, 836	4.7 [3, 6] 656 383, 836	3.7 [2, 5] 529 288, 720	3.9 [3, 5] 563 396, 720	3.2 [2, 4] 429 271, 542	2.8 [1, 4] 374 169, 542	5.0 [2, 8] 722 290, 1158	4.0 [2, 6] 585 290, 869	[0, 1] 219 0, 212	0.9 [0, 1] 180 0, 212	2.7 [1, 4] 374 138, 550	2.9 [1, 4] 393 138, 550
Venepuncture blood sampling - Average number (IQR) - Average costs per patient - IQR of average costs	17.7 [7, 23] 950 376, 1223	17.0 [8, 223] 915 430, 1237	30.6 [18, 39] 1923 1102, 2455	32.9 [19, 42] 2073 1165, 2628	13.7 [7, 20] 276 136, 403	12.1 [4, 19] 244 81, 383	17.4 [7, 22] 3229 1299, 3991	16.4 [9, 23] 3050 1671, 4223	4.2 [0, 6] 20 0, 30	4.7 [2, 6] 23 11, 30	10.8 [0, 16] 25 0, 37	17.9 [6, 27] 41 14, 62
Radiation therapy - Average number of days (IQR) - Average costs per patient - IQR of average costs	4.6 [0, 1] 19,553 0, 3200	1.5 [0, 0] 6437 0, 0	3.9 [0, 6] 9303 0, 13,640	2.7 [0, 2] 6326 0, 4151	2.1 [0, 0] 105 0, 0	1.9 [0, 0] 95 0, 0	0.9 [0, 0] 3421 0, 0	0.6 [0, 0] 2197 0, 0	0 0	0.3 [0, 0] 370 0, 0	0.8 [0, 0] 121 0, 0	0.6 [0, 0] 103 0, 0
Immunotherapy - Average number of days (IQR) - Average costs per patient - IQR of average costs	1.6 [0, 0] 5621 0, 0	2.5 [0, 0] 8483 0, 0	2.5 [0, 5] 9918 0, 18,132	8.7 [0, 16] 35,104 0, 64,469	3.1 [0, 0] 9539 0, 0	1.9 [0, 0] 5663 0, 0	1.7 [0, 0] 5810 0, 0	4.9 [0, 7] 17,099 0, 24,400	0.1 [0, 0] 210 0, 0	0 0	0.9 [0, 0] 3134 0, 0	0.1 [0, 0] 621 0, 0
Antibiotics - Average number of days (IQR) - Average costs per patient - IQR of average costs	5.9 [0, 9] 80 0, 123	6.6 [0, 9] 90 0, 123	21.8 [3, 14] 347 0, 227	28.1 [2, 26] 448 32, 415	6.5 [0, 10] 86 0, 133	1.0 [0, 0] 14 0, 0	2.1 [0, 0] 16 0, 0	1.9 [0, 0] 14 0, 0	2.6 [0, 0] 18 0, 0	5.5 [0, 10] 37 0, 68	4.4 [0, 5] 101 0, 116	4.8 [0, 7] 112 0, 162
*Total mean healthcare costs* *IQR mean healthcare costs*	*54,896* *20,466, 81,587*	*48,529* *21,156, 59,622*	*61,177* *31,735, 74,941*	*79,901* *26,040, 127,853*	*41,848* *14,195, 61,291*	*36,255* *6101, 29,473*	*25,361* *7158, 35,733*	*36,448* *12,584, 48,135*	*6068* *0, 9325*	*5761* *365, 8800*	*169,38* *4192, 20,354*	*28,232* *5671, 27,019*

# Based on real country-specific unit prices and, if those were not available, on average unit prices of the other countries

a *Due to unknown content of specialist palliative care services costs could not be established.*

For most categories, the use of care was not statistically different between study groups. For instance, the average length of hospital stays was 9 days in the intervention group versus 8 in the control group (*p* = 0.07), mean use of radiotherapy was 2.0 versus 1.2 days (*p* = 0.22), and 5 participants in the intervention group (1.4%) received cardiopulmonary resuscitation versus 3 control participants (0.5%) (*p* = 0.14). In other categories, use of care was statistically different, for instance in the intervention group compared to the control group the mean numbers of MRI scans were 0.6 versus 0.3 (*p*< 0.001), mean use of immunotherapy was 1.5 versus 3.1 days (*p* = 0.001), and specialist palliative care services were used by 36.7 versus 27.4% of patients (*p* = 0.002).

In addition to differences between study groups, we observed differences in use of care and costs per country, see Table 3. For instance, in Belgium the mean number of hospital days and of chemotherapy days was higher than in the other countries. Also, in Belgium the mean number of hospital days and of intravenous chemotherapy days was higher in intervention patients than control patients (*p* = 0.011 and *p* = 0.013) while in the Netherlands and the UK, the opposite was observed. Further, the proportions of patients included in the intervention versus control group differed per country.

Country, religion, and WHO performance status were significantly associated with total costs in univariate multilevel GLMM models. The multivariable GLMM multilevel model, which included all variables that had been considered in the univariate GLMMs, showed lower average costs of care in the intervention group, related to differences between study groups in country, religion and WHO-status, see Table 4. No effect of the intervention on differences in costs between study groups was observed (*p* = 0.3).

**Table 4.** Associations of characteristics of patients with total costs of care.

	Unvariable Multivariable					
Characteristic	exp(Beta)	95% CI	*p*-Value	exp(Beta)	95% CI	*p*-Value
Study group						
Control group	Ref					
Intervention group	0.75	0.41, 1.38	0.4	0.91	0.76, 1.10	0.3
Age_category						
18–45 years	Ref					
46–65 years	0.88	0.53, 1.46	0.6	0.85	0.50, 1.43	0.5
>65 years	0.89	0.54, 1.46	0.6	0.85	0.50, 1.43	0.5
Sex						
Male	Ref					
Female	0.89	0.75, 1.05	0.2	0.87	0.72, 1.04	0.12
Country						
The Netherlands	Ref					
Belgium	1.54	1.19, 1.99	0.001	1.56	1.19, 2.05	0.001
Slovenia	0.18	0.13, 0.26	<0.001	0.21	0.14, 0.30	<0.001
Italy	0.96	0.73, 1.27	0.8	0.85	0.63, 1.15	0.3
Denmark	2.08	1.56, 2.76	<0.001	2.11	1.56, 2.85	<0.001
United Kingdom	0.73	0.58, 0.93	0.012	0.75	0.58, 0.96	0.023
Religious						
Yes	Ref					
No	0.83	0.69, 1.00	0.053	0.82	0.68, 0.99	0.043
Prefer not to specify	0.65	0.50, 0.84	0.001	0.70	0.54, 0.92	0.009
Cancer type						
Small cell-lung cancer	Ref					
Non-small cell lung cancer	1.34	0.99, 1.80	0.055	1.25	0.92, 1.70	0.15
Colon cancer	1.22	0.89, 1.67	0.2	1.33	0.39, 4.50	0.6
Rectal cancer	1.36	0.93, 1.97	0.11	1.51	0.45, 5.08	0.5
Current_stage						
Stage III, lung cancer	Ref					
Stage IV, lung cancer	1.07	0.81, 1.41	0.6	1.02	0.77, 1.34	>0.9
Colorectal cancer stage IV	0.96	0.72, 1.28	0.8	0.73	0.22, 2.44	0.6
Colorectal cancer – metachronous metastases	1.23	0.86, 1.78	0.3	0.96	0.28, 3.29	>0.9
WHO performance status						
0 Fully active	Ref					
1 No heavy physical work	0.79	0.65, 0.96	0.019	0.85	0.70, 1.04	0.11
2 Up for more than half the day	0.69	0.51, 0.94	0.019	0.77	0.57, 1.03	0.078
3 In bed/sitting more than half the day	0.27	0.14, 0.55	<0.001	0.37	0.18, 0.76	0.007

The sensitivity analyses, in which data of patients who died during follow-up were excluded, showed similar results: lower average costs of care in the intervention group, related to differences between study groups in country, religion and WHO-status, and no effect of the intervention on differences in costs between study groups was observed, see Supplemental Appendix 4.

Bootstrap analyses to establish whether advance care planning was associated with lower costs in individual countries showed that this was the case in the Netherlands (*p* = 0.008). An overview of costs of ACP conversations and total costs of use of care in the intervention group by country can be found in Supplemental Appendix 5.

## Discussion

### Main findings of the study

This is the first randomised controlled trial to investigate the impact of advance care planning on healthcare costs in patients with advanced cancer in Europe. We found that the use and costs of hospital care were lower in the advance care planning intervention group. These reduced costs are at least partly attributed to differences in patient characteristics between the intervention and control groups, such as age, country of residence, being religious, type of cancer, and WHO performance status. The complete-case analysis showed no association between the intervention and costs. A definite impact of the intervention could therefore not be established based on our findings. Advance care planning has previously been found to be associated with reduced healthcare use and costs,^6–9,11–13,15,17,18,21,23,24,30^ which may be related to adults choosing less or less invasive medical interventions after engaging in advance care planning. Two of these studies had a randomised design^7,13^ one focused at nursing home populations^7^ and one at in-patient populations.^13^

We observed that survival at 12 months follow-up was significantly lower in the intervention group (*p* = 0.01). In addition, patients with a good performance status more often received systemic treatment and their care costs were higher, irrespective of whether they received the advance care planning intervention or not. However, they were underrepresented in the intervention group.

We found that healthcare use and cost patterns differed per country, with higher costs in Denmark and the United Kingdom. Differences in length of hospital stay per country, as observed in our study, are potentially related to a range of circumstances, such as differences in organisation of care^37^ and availability of home care facilities. Studies showed that substantial differences of expenditures between countries were caused by local variations in the approach to the management of patients receiving palliative care in terms of hospitalisations and diagnostics.^38,39^ Among patients older than 65 years who died with cancer in 2010, end-of-life care was more hospital-centric in, for instance, Belgium than in the Netherlands.^39^ We also know that at the start of the study, the concept of ACP was almost unknown in Denmark, Italy and Slovenia, and ADs had no legal status in Italy. Still, understanding of variations in health expenditures for patients with cancer in Europe needs to improve.^40^

### Strengths and limitations of the study

Strengths of this study include its international character, its randomised controlled design and the high rate of intervention group participants who completed the advance care planning programme. In addition, we were able to include core cost categories (hospital care, diagnostic procedures, medical interventions and medication) in our economic evaluation. Our study has some limitations as well. In some countries, more intervention than control patients were included while in others this was the other way around. We do not expect this hugely biased our estimates since the inclusion criteria were similar in all countries and we purposefully defined them objectively, using terms of the TNM classification and staging system. However, given that usual care may differ between countries, for instance more or less routine discussions about care preferences, this may have impacted the contrast between intervention and control group. Also, there is the possibility of professional gatekeeping and that patients who were already more oriented towards advance care planning more often agreed to take part in the ACTION study, as far as it concerns the intervention group. We hypothesise that participating in advance care planning may have been less appealing to patients who were focused at cure. Therefore those invited to participate in the intervention group may have more often declined than those invited to participate in the control group. Lower response rates in intervention hospitals may have resulted in a selection bias, since in the intervention group fewer patients than in the control group received systemic therapy (79.2 vs 88.9%, respectively) and fewer had WHO performance status 0 (fully active) than in the control group (24.9 vs 39.8%, see Table 1). Patients with a suboptimal performance status who no longer receive systemic therapy may weigh the pros and cons of using hospital services or the trade-off between length and quality of life differently. Analyses of medical care were limited to retrospective data-collection in the files of the hospital where patients were included in the study. Thus, if patients were also seen elsewhere, information about hospital care may not have been complete. Also, we were able to conduct analyses of hospital files for 365/442 (83%) patients in the intervention group and 583/675 (86%) patients in the control group, meaning that 15% of files have not been analysed. The percentage of complete cases was 83%. Further, we did not collect data on costs of care people may have received at home, from other healthcare institutions, or costs of involvement of informal caregivers. Findings have to be interpreted with caution, given the relatively low number of hospital stays, diagnostic procedures, and medical interventions. Not all unit prices were available for all countries, and in such cases unit prices had to be based on average unit prices of the other countries, and corrected for the purchasing power parities (PPP) for general domestic product (GDP) per country. This may have resulted in inaccuracies, such as smaller standard errors due to reduced variation. However, due to the correction per country of the mean prices, we believe the reduction in standard errors is limited.

### What this study adds

Studying the effects of advance care planning in a cohort of patients with advanced disease is challenging, but given the interest in the implementation of advance care planning programmes and the limited available knowledge about the effects in such cohorts, it is essential to study these effects and to provide much needed insight. While costs should not be the primary consideration in offering advance care planning, cost studies about advance care planning provide essential additional information for healthcare organisations who consider implementation of advance care planning programmes. We found lower costs of care in the intervention group, but established that this was mainly explained by differences in patient characteristics between the groups.

We recommend that future research prospectively monitors the use of care during the full trajectory from advance care planning conversations until the patient’s death to be able to measure the full impact of advance care planning on costs of hospital care. Further, future research might apply a broader perspective and include, for instance, nursing and home care.

## Supplemental Material

sj-pdf-1-pmj-10.1177_02692163221142950 – Supplemental material for Healthcare use and healthcare costs for patients with advanced cancer; the international ACTION cluster-randomised trial on advance care planning Click here for additional data file. Supplemental material, sj-pdf-1-pmj-10.1177_02692163221142950 for Healthcare use and healthcare costs for patients with advanced cancer; the international ACTION cluster-randomised trial on advance care planning by Ida J Korfage, Suzanne Polinder, Nancy Preston, Johannes JM van Delden, A)JLM Geraerds, Lesley Dunleavy, Kristof Faes, Guido Miccinesi, Giulia Carreras, Caroline Moeller Arnfeldt, Marijke C Kars, Giuseppe Lippi, Urska Lunder, Ceu Mateus, Kristian Pollock, Luc Deliens, Mogens Groenvold, Agnes van der Heide and Judith AC Rietjens in Palliative Medicine

sj-pdf-2-pmj-10.1177_02692163221142950 – Supplemental material for Healthcare use and healthcare costs for patients with advanced cancer; the international ACTION cluster-randomised trial on advance care planning Click here for additional data file. Supplemental material, sj-pdf-2-pmj-10.1177_02692163221142950 for Healthcare use and healthcare costs for patients with advanced cancer; the international ACTION cluster-randomised trial on advance care planning by Ida J Korfage, Suzanne Polinder, Nancy Preston, Johannes JM van Delden, A)JLM Geraerds, Lesley Dunleavy, Kristof Faes, Guido Miccinesi, Giulia Carreras, Caroline Moeller Arnfeldt, Marijke C Kars, Giuseppe Lippi, Urska Lunder, Ceu Mateus, Kristian Pollock, Luc Deliens, Mogens Groenvold, Agnes van der Heide and Judith AC Rietjens in Palliative Medicine

sj-pdf-3-pmj-10.1177_02692163221142950 – Supplemental material for Healthcare use and healthcare costs for patients with advanced cancer; the international ACTION cluster-randomised trial on advance care planning Click here for additional data file. Supplemental material, sj-pdf-3-pmj-10.1177_02692163221142950 for Healthcare use and healthcare costs for patients with advanced cancer; the international ACTION cluster-randomised trial on advance care planning by Ida J Korfage, Suzanne Polinder, Nancy Preston, Johannes JM van Delden, A)JLM Geraerds, Lesley Dunleavy, Kristof Faes, Guido Miccinesi, Giulia Carreras, Caroline Moeller Arnfeldt, Marijke C Kars, Giuseppe Lippi, Urska Lunder, Ceu Mateus, Kristian Pollock, Luc Deliens, Mogens Groenvold, Agnes van der Heide and Judith AC Rietjens in Palliative Medicine

sj-pdf-4-pmj-10.1177_02692163221142950 – Supplemental material for Healthcare use and healthcare costs for patients with advanced cancer; the international ACTION cluster-randomised trial on advance care planning Click here for additional data file. Supplemental material, sj-pdf-4-pmj-10.1177_02692163221142950 for Healthcare use and healthcare costs for patients with advanced cancer; the international ACTION cluster-randomised trial on advance care planning by Ida J Korfage, Suzanne Polinder, Nancy Preston, Johannes JM van Delden, A)JLM Geraerds, Lesley Dunleavy, Kristof Faes, Guido Miccinesi, Giulia Carreras, Caroline Moeller Arnfeldt, Marijke C Kars, Giuseppe Lippi, Urska Lunder, Ceu Mateus, Kristian Pollock, Luc Deliens, Mogens Groenvold, Agnes van der Heide and Judith AC Rietjens in Palliative Medicine

sj-pdf-5-pmj-10.1177_02692163221142950 – Supplemental material for Healthcare use and healthcare costs for patients with advanced cancer; the international ACTION cluster-randomised trial on advance care planning Click here for additional data file. Supplemental material, sj-pdf-5-pmj-10.1177_02692163221142950 for Healthcare use and healthcare costs for patients with advanced cancer; the international ACTION cluster-randomised trial on advance care planning by Ida J Korfage, Suzanne Polinder, Nancy Preston, Johannes JM van Delden, A)JLM Geraerds, Lesley Dunleavy, Kristof Faes, Guido Miccinesi, Giulia Carreras, Caroline Moeller Arnfeldt, Marijke C Kars, Giuseppe Lippi, Urska Lunder, Ceu Mateus, Kristian Pollock, Luc Deliens, Mogens Groenvold, Agnes van der Heide and Judith AC Rietjens in Palliative Medicine

sj-pdf-6-pmj-10.1177_02692163221142950 – Supplemental material for Healthcare use and healthcare costs for patients with advanced cancer; the international ACTION cluster-randomised trial on advance care planning Click here for additional data file. Supplemental material, sj-pdf-6-pmj-10.1177_02692163221142950 for Healthcare use and healthcare costs for patients with advanced cancer; the international ACTION cluster-randomised trial on advance care planning by Ida J Korfage, Suzanne Polinder, Nancy Preston, Johannes JM van Delden, A)JLM Geraerds, Lesley Dunleavy, Kristof Faes, Guido Miccinesi, Giulia Carreras, Caroline Moeller Arnfeldt, Marijke C Kars, Giuseppe Lippi, Urska Lunder, Ceu Mateus, Kristian Pollock, Luc Deliens, Mogens Groenvold, Agnes van der Heide and Judith AC Rietjens in Palliative Medicine
