# Supplementary material for: Healthcare use and healthcare costs for patients with advanced cancer; the international ACTION cluster-randomised trial on advance care planning
Source: Palliat Med. 2022 Dec 14;37(5):707–18. doi: 10.1177/02692163221142950 (PMC10227094; doi:10.1177/02692163221142950)
Supplement: sj-pdf-1-pmj-10.1177_02692163221142950 – Supplemental material for Healthcare use and healthcare costs for patients with advanced cancer; the international ACTION cluster-randomised trial on advance care planning [file sj-pdf-1-pmj-10.1177_02692163221142950.pdf]

Box 1: Costs of ACP conversations performed by nurse or medical specialist

	Unit price [Min, Max](€)[a]	Average Quantity Mean [IQR]	Average total costs (€) Mean [IQR]
Nurse	[16, 52]	1.5 [1, 2]	49 [29, 66]
Medical specialist	[32, 122]	1.5 [1, 2]	117 [58, 161]

[a] Price per hour for ACP conversation; Minimum and Maximum price indicate lowest price and highest price for different countries
