# Supplementary material for: Healthcare use and healthcare costs for patients with advanced cancer; the international ACTION cluster-randomised trial on advance care planning
Source: Palliat Med. 2022 Dec 14;37(5):707–18. doi: 10.1177/02692163221142950 (PMC10227094; doi:10.1177/02692163221142950)
Supplement: sj-pdf-2-pmj-10.1177_02692163221142950 – Supplemental material for Healthcare use and healthcare costs for patients with advanced cancer; the international ACTION cluster-randomised trial on advance care planning [file sj-pdf-2-pmj-10.1177_02692163221142950.pdf]

Appendix 1: Unit prices of healthcare items in 2018 €

**Healthcare item**	**Netherlands**	**Belgium**	**Italy**	**Slovenia**	**UK[a]**	**Denmark[b]**
Hospitalisation						
Hospital day	458.21	452.41	400.21	330.61	458.88	Only academic
Hospital day academic	664.04	655.64	579.99	479.12	665.01	767.60
ICU day	1,226.72	1,211.19	1,071.44	885.10	1,228.51	1,418.02
Diagnostics						
Ultrasound	87.92	52.41	52.59	39.67	79.39	70.00
MRI	223.42	143.73	341.35	177.02	197.22	312.00
PET scan	945.00	176.45	859.69	450.49	389.84	721.74
CT scan	144.81	139.27	135.53	212.28	137.59	144.00
X-ray	56.89	29.71	39.95	13.57	34.40	67.00
Bone scan	200.06	185.68	139.07	139.85	228.17	224.05
Venepuncture for lab	185.62	53.77	20.15	4.93	2.29	62.95
Endoscopy	465.45	195.46	117.32	162.08	40.13	594.00
Bronchoscopy	184.00	142.48	107.21	146.00	254.55	2,202.00
Biopsy	126.00	84.78	49.72	65.49	322.19	223.00
Medical intervention						
IV therapy	1,400.00	1,382.28	1,222.78	1,010.13	1,402.05	1,618.33

Oral chemotherapy	215.00	307.94	380.00	225.03	195.78	360.53
Radiation therapy	3670.00	4266.00	50.22	1480.66	160.51	2372.16
Immunotherapy	3485.70	3441.58	3044.47	2515.00	3490.79	4029.29
Targeted therapy	294.64	954.59	2276.00	69.98	968.24	1117.61
General treatment						
Cardiopulmonary resuscitation	270.82	267.39	236.54	195.40	271.21	313.05
Artificial nutrition	200.00	236.37	209.09	45.55	455.48	276.73
Artificial hydration	11.12	10.97	9.71	8.02	11.13	12.85
Medication						
Antibiotics	7.50	13.63	13.30	6.80	23.10	15.96

NOTE: Grey fields indicate that prices were unavailable for this country, and are therefore based on costs of other countries, corrected for GDP

[a]Exchange rate Pound to €: 1.147

[b]Exchange rate Danish Krone to €: 0.134
