# Supplementary material for: Healthcare use and healthcare costs for patients with advanced cancer; the international ACTION cluster-randomised trial on advance care planning
Source: Palliat Med. 2022 Dec 14;37(5):707–18. doi: 10.1177/02692163221142950 (PMC10227094; doi:10.1177/02692163221142950)
Supplement: sj-pdf-3-pmj-10.1177_02692163221142950 – Supplemental material for Healthcare use and healthcare costs for patients with advanced cancer; the international ACTION cluster-randomised trial on advance care planning [file sj-pdf-3-pmj-10.1177_02692163221142950.pdf]

Appendix 2: Univariable Generalized linear model for total healthcare costs

Variable	Exp(E) (95% CI)	P-value
Intervention group	-0.189 (-0.344, -0.035)	0.016*
Age		
18-45y	Ref	
45-65y	-0.577 (-1.019, -0.135)	0.011*
65y+	-0.665 (-1.104, -0.227)	0.003*
Female gender	-0.119 (-0.272, 0.034)	0.127
Religion		
Religious	Ref	
Not religious	-0.109 (-0.272, 0.054)	0.189
Prefers not to specify	-0.268 (-0.503, -0.033)	0.025*
Diagnosis		
Small cell lung cancer	Ref	
Non-small cell lung cancer	0.387 (0.131, 0.644)	0.003*
Colon cancer	0.325 (0.061, 0.588)	0.016*
Rectal cancer	0.339 (0.019, 0.659)	0.038*
Stage of cancer		
Stage III lung cancer	Ref	
Stage IV lung cancer	-0.038 (-0.289, 0.213)	0.765
Stage IV colorectal cancer	-0.177 (-0.438, 0.084)	0.184
Metachronous metastases colorectal cancer	0.246 (-0.056, 0.548)	0.111
WHO performance status		
Fully active	Ref	
No heavy physical work	-0.277 (-0.277, -0.277)	<0.001*
Up for more than half of the day	41.634 (41.634, 41.634)	<0.001*
In bed/sitting more than half of the day	137.106 (137.106, 137.106)	<0.001*
