# Supplementary material for: Healthcare use and healthcare costs for patients with advanced cancer; the international ACTION cluster-randomised trial on advance care planning
Source: Palliat Med. 2022 Dec 14;37(5):707–18. doi: 10.1177/02692163221142950 (PMC10227094; doi:10.1177/02692163221142950)
Supplement: sj-pdf-4-pmj-10.1177_02692163221142950 – Supplemental material for Healthcare use and healthcare costs for patients with advanced cancer; the international ACTION cluster-randomised trial on advance care planning [file sj-pdf-4-pmj-10.1177_02692163221142950.pdf]

ACTION

DATA EXTRACTION FORM FOR REVIEW OF HOSPITAL MEDICAL RECORDS & INSTRUCTIONS FOR COMPLETION

ACTION –DATA EXTRACTION FORM FOR REVIEW OF HOSPITAL MEDICAL RECORDS & INSTRUCTIONS FOR COMPLETION

	**INSTRUCTIONS FOR COMPLETION**
**Please complete before assessing/checking the patient's hospital medical records:**<br>Name reviewer: ....<br>Study number of patient: ....<br>Hospital: ....<br>Date of inclusion of patient in ACTION: …../…../…..<br>Date of completion of data extraction form: …../…../…..	This section must be completed before assessing the patient's hospital medical records.<br><br>Date of inclusion in ACTION is the date on which the patient signed the informed consent form.

**ITEMS TO BE EXTRACTED**		**INSTRUCTIONS FOR COMPLETION**
**Please complete based on the patient's hospital medical records only:**		All items must be completed based on the patient's <u>hospital medical records only</u>. Other information must not be taken into account.<br><br>The review of hospital medical records relates to the <u>12 months post inclusion</u> or, in cases where the patient died within 12 months after inclusion, the time between inclusion and the patient's death.
**SURVIVAL**		
1. Did the patient die within 12 months following inclusion in ACTION?	❒ Yes – ***Go to question 2***<br>❒ No – ***Go to question 4***<br>❒ Unknown/ information not available in records - ***Go to question 4***	Date of inclusion in ACTION is the date on which the patient signed the informed consent form.
**DATE AND PLACE OF DEATH**		
2. What was the date of death?	.... / .... / ....<br>❒ Unknown/ information not available in records	All items must be completed based on the patient's hospital medical records only. Other information must not be taken into account.
3. What was the place of death?	❒ Home<br>❒ Hospital – ward<br>❒ Hospital – intensive care unit<br>❒ Hospital – emergency department<br>❒ Long term care setting<br>❒ Palliative care setting (e.g. hospital palliative care unit, hospice)<br>❒ Other place of death, specify ....<br>..<br>..<br>..<br>..<br>❒ Unknown/ information not available in records	All items must be completed based on the patient's hospital medical records only. Other information must not be taken into account.<br><br>Palliative care setting: institution for inpatient specialist palliative care, e.g. hospital palliative care unit, hospice.

ADVANCE DIRECTIVES		
4. Does the medical file contain a completed copy of the My Preferences form (MPF) of the ACTION study?	❒ Yes – Date first documented: …../…../…..; Date last documented:…../…../….. - ***Go to question 5***<br>❒ No – ***Go to question 10***	Questions 4 to 9 refer to the content of the last documented My Preferences Form (MPF), that may be found in the patient's hospital medical records.<br><br>Specify the dates on which the MPF was documented for the first and the last time.<br><br>In order to find a copies of the MPF, at least all scanned documents in the hospital medical records are to be examined over the 12 months post inclusion or, in case the patient dies within 12 months after inclusion, over the time between inclusion and the patient's death. Also admission notes and other types of notes may be searched.
5. Did the patient assign someone as personal representative?	❒ Yes<br>❒ No	The personal representative is a person that was asked by the patient to express the patient's preferences for care and treatments so they can be taken into account when the patient is unable to make his/her own decisions.<br><br>See the lower part of page 3 of MPF.
6. What was the preference with regard to cardiopulmonary resuscitation (CPR) (section C of the MPF)?	❒ To have CPR attempted<br>❒ Not to have CPR attempted<br>❒ Section C was not completed or preference was unclear	See section C on page 5 of MPF.
7. What was the preference with regard to the goals of future care (Section D of the MPF)?	❒ Selective Treatment plus Comfort-focused Care<br>❒ Comfort-focused Care<br>❒ Section D was not completed or preference was unclear	See section D on page 5 of MPF.
8. What was the preference with regard to the final place of care (Section E of the MPF)?	❒ The patient had a preferred final place of care, namely:<br>❒ Home<br>❒ Long term care setting<br>❒ Palliative care setting (e.g. hospital palliative care unit, hospice)<br>❒ Hospital<br>❒ Other, specify ……………………………………………………….<br>……………………………………………………………………….<br>……………………………………………………………………….<br>❒ The patient did not have a preferred final place of care<br>❒ Section E was not completed or preference was unclear	See section E on page 6 of MPF.

9. Were there other preferences regarding future care and treatments (Section F of the MPF) *(More than 1 option possible)*?	❒ Intubation:<br>❒ Do intubate for mechanical ventilation<br>❒ Do not intubate for mechanical ventilation<br>❒ Hospitalisation:<br>❒ Do hospitalise<br>❒ Do not hospitalise<br>❒ Admission to intensive care unit:<br>❒ Do admit to intensive care unit<br>❒ Do not admit to intensive care unit<br>❒ Artificial nutrition:<br>❒ Do provide artificial nutrition and hydration<br>❒ Do not provide artificial nutrition and hydration<br>❒ Antibiotics:<br>❒ Do provide antibiotics<br>❒ Do not provide antibiotics<br>❒ Request for physician assistance in dying (e.g. euthanasia)<br>❒ No treatment limitations<br>❒ Other, specify ....<br>...<br>...<br>...<br>...	See section F on page 6 of MPF.<br><br>Not ticking the box for 'Intubation', 'Hospitalisation', etc. (leftmost boxes), means there was no expressed preference for intubation, hospitalisation, etc.<br><br>Potential answer only in Belgium and the Netherlands.
10. Does the medical file contain a completed copy of an advance directives form other than the My Preferences form?	❒ Yes – Date first documented: …../…../…..; Date last documented:…../…../….. - ***Go to question 11***<br>❒ No – ***Go to question 14***	Questions 10 to 13 refer to the content of the last documented advance directives form other than the MPF. All preferences expressed in the advance directives form should be recorded, regardless of whether they were also expressed in the MPF. E.g., when preferences were stated in the MPF ánd in another advance directives form, they have to be recorded again.<br><br>Specify the dates on which the advance directives form was documented for the first and the last time.<br><br>An advance directives form, also called living will or advance decision, is a patient written and/or patient signed document in which a person specifies preferences and decisions about future treatments and care.<br><br>In order to find a copies of an advance directives form, at least all scanned documents in the hospital medical records are to be examined over the 12 months post inclusion or, in case the patient dies within 12 months after inclusion, over the time between inclusion and the patient's death. Also admission notes and other types of notes may be searched.

11. Did the patient assign someone as personal representative?	❒ Yes<br>❒ No	The personal representative is a person that was asked by the patient to express the patient's preferences for care and treatments so they can be taken into account when the patient is unable to make his/her own decisions.<br><br>The authorization of personal representative or agent, may be done in a separate document, e.g. a lasting power of attorney form. At least all scanned documents in the hospital medical records are to be examined over the 12 months post inclusion or, in case the patient dies within 12 months after inclusion, over the time between inclusion and the patient's death. Also admission notes and other types of notes may be searched.
12. Did the patient indicate a preference regarding the final place of care?	❒ Yes, the patient indicated a preferred final place of care, namely:<br>❒ Home<br>❒ Long term care setting<br>❒ Palliative care setting (e.g. hospital palliative care unit, hospice)<br>❒ Hospital<br>❒ Other, specify ....<br>..<br>..<br>❒ No, the patient did not indicate a preferred final place of care<br>❒ Unclear	
13. About which topics regarding future care and treatments were preferences included, and which preferences *(More than 1 option possible)*?	❒ Resuscitation:<br>❒ Do resuscitate<br>❒ Do <u>not</u> resuscitate<br>❒ Intubation:<br>❒ Do intubate for mechanical ventilation<br>❒ Do <u>not</u> intubate for mechanical ventilation<br>❒ Hospitalisation:<br>❒ Do hospitalise<br>❒ Do <u>not</u> hospitalise<br>❒ Admission to intensive care unit:<br>❒ Do admit to intensive care unit<br>❒ Do <u>not</u> admit to intensive care unit<br><br>❒ Artificial nutrition:<br>❒ Do provide artificial nutrition and hydration<br>❒ Do <u>not</u> provide artificial nutrition and hydration<br>❒ Antibiotics:<br>❒ Do provide antibiotics<br>❒ Do <u>not</u> provide antibiotics<br>❒ No treatment limitations<br>❒ Request for physician assistance in dying (e.g. euthanasia)<br>❒ Other, specify ....<br>..<br>..	Not ticking the box for 'Resuscitation, 'Intubation', etc. (leftmost boxes), means there was no expressed preference for resuscitation, intubation, etc.<br><br>Potential answer only in Belgium and the Netherlands.

	……………………………………………………………………………………<br>……………………………………………………………………………………	
14. Does the medical file contain preferences for future care and treatments that were expressed orally and noted in the hospital medical record, e.g. in physician notes or in notes of a palliative care team?	❒ Yes – Date first documented: …../…../….. ; Date last documented:…../…../…..- ***Go to question 15***<br>❒ No – ***Go to question 18***	Questions 14 to 17 refer to the content of the last documented patient preferences for future care and treatments that were expressed orally by the patient and subsequently noted in the patient's hospital medical record, e.g. in physician notes or in notes of a palliative care team. All preferences expressed orally and noted in the hospital medical records should be recorded, regardless whether they were also expressed in the MPF or any other advance directive form. E.g., when preferences were stated in the MPF ánd expressed orally (and subsequently noted in the hospital medical records), they have to be recorded again.<br><br>Specify the date on which orally expressed preferences for future care and treatments were documented for the first and the last time.<br><br>In order to retrieve orally expressed preferences for future care and treatments, various types of notes will have to be searched, at least including: physician notes, notes of a palliative care team.
15. Did the patient orally assign someone as personal representative?	❒ Yes<br>❒ No	The personal representative is a person that was asked by the patient to express the patient's preferences for care and treatments so they can be taken into account when the patient is unable to make his/her own decisions.<br><br>In order to retrieve whether the patients assigned orally a personal representative, various types of notes will have to be searched, at least including: physician notes, notes of a palliative care team.
16. Did the patient orally indicate a preference regarding the final place of care?	❒ Yes, the patient indicated a preferred final place of care, namely:<br>❒ Home<br>❒ Long term care setting<br>❒ Palliative care setting (e.g. hospital palliative care unit, hospice)<br>❒ Hospital<br>❒ Other, specify …………………………………………………………<br>……………………………………………………………………………<br>……………………………………………………………………………<br>❒ No, the patient did not indicate a preferred final place of care<br>❒ Unclear	

17. About which topics regarding future treatments did the patient orally express preferences, and which preferences *(More than 1 option possible)*?	❒ Resuscitation:<br>❒ Do resuscitate<br>❒ Do not resuscitate<br>❒ Intubation:<br>❒ Do intubate for mechanical ventilation<br>❒ Do not intubate for mechanical ventilation<br>❒ Hospitalisation:<br>❒ Do hospitalise<br>❒ Do not hospitalise<br>❒ Admission to intensive care unit:<br>❒ Do admit to intensive care unit<br>❒ Do not admit to intensive care unit<br><br>❒ Artificial nutrition:<br>❒ Do provide artificial nutrition and hydration<br>❒ Do not provide artificial nutrition and hydration<br>❒ Antibiotics:<br>❒ Do provide antibiotics<br>❒ Do not provide antibiotics<br>❒ Request for physician assistance in dying (e.g. euthanasia)<br>❒ No treatment limitations<br>❒ Other, specify ....<br>..<br>..<br>..<br>..	Not ticking the box for 'Resuscitation, 'Intubation', etc. (leftmost boxes), means there was no expressed preference for intubation, hospitalisation, etc.<br><br>Potential answer only in Belgium and the Netherlands.
**PHYSICIAN ORDERS**		
18. Does the medical file contain physician orders for future treatments and care?	❒ Yes – Date first documented: …../…../…..; Date last documented:…../…../….. -***Go to question 19***<br>❒ No – ***Go to question 21***	Questions 18 to 20 relate to the content of the last documented physician written and/or physician signed orders for future treatments and care.<br><br>Specify the dates on which the physician orders for treatment were documented for the first and the last time.<br><br>To document physician orders, a specific form may have been used. However, physician orders may also be noted in physician notes.<br><br>In order to find physician orders, at least all scanned documents ànd physician notes in the hospital medical records are to be examined over the 12 months post inclusion or, in case the patients dies within 12 months after inclusion, over the time between inclusion and the patient's death.

19. About which topics regarding future treatments were physician orders included, and which orders *(More than 1 option possible)*?	❒ Resuscitation:<br>❒ Do resuscitate<br>❒ Do not resuscitate<br>❒ Admission to intensive care unit:<br>❒ Do admit to intensive care unit<br>❒ Do not admit to intensive care unit<br>❒ Intubation:<br>❒ Do intubate for mechanical ventilation<br>❒ Do not intubate for mechanical ventilation<br>❒ Artificial nutrition and hydration:<br>❒ Do provide artificial nutrition and hydration<br>❒ Do not provide artificial nutrition and hydration<br>❒ Antibiotics:<br>❒ Do provide antibiotics<br>❒ Do not provide antibiotics<br>❒ No treatment limitations<br>❒ Other, specify ....<br>..<br>..<br>..<br>..	Question 19 refers to phyiscian orders about future treatments, not about current treatment. E.g. it is of interest to know whether or not a decision was taken by a physician to provide antibiotics in the future, not whether the physician currently prescribes antibiotics.<br><br>Not ticking the box for 'Resuscitation', 'Admission to intensive care unit', etc. (leftmost boxes), means there were no physician orders for resuscitation, admission to intensive care unit, etc.
20. With whom were these physician orders discussed? *(More than 1 option possible)*	❒ Patient<br>❒ Personal representative<br>❒ Relative<br>❒ Other professional caregiver<br>❒ Other, specify ....<br>..<br>..<br>..<br>❒ No one<br>❒ Unclear	Specific forms for physician orders, physician notes, as well as other notes in the hospital records may contain information on whether the physician orders have been discussed with patients, their personal representative, relatives or other health care professionals.
**DIAGNOSTIC PROCEDURES AND TREATMENTS**		
21. Do the medical records indicate that diagnostic procedures were used during the 12 months following inclusion or until death?	❒ Yes – ***Go to question 22***<br>❒ No – ***Go to question 23***	Questions 21 to 25 refer to diagnostic procedures and treatments that the patient received in hospital either as an outpatient or as an inpatient in the 12 months following inclusion or untill the patient's death in case the patients dies within 12 months after inclusion.<br><br>In order to find information about diagnostic procedures and treatments, various types of notes will have to be searched, at least including: physician notes, orders for diagnostic procedures, orders for the administration of drugs and therapies, test results, reports, admission notes, progress notes, etc.
22. Which diagnostic procedures?	❒ Ultrasound .... times<br>❒ MRI scan .... times	

	❒ PET scan .... times<br>❒ CT scan .... times<br>❒ X-ray .... times<br>❒ Bone scan (scintigram) .... times<br>❒ Venipuncture for blood sampling .... times<br>❒ Endoscopy .... times<br>❒ Bronchoscopy .... times<br>❒ Biopsy .... times	It is sufficient to count the number of venipunctures for blood sampling. It is not necessary to count the number of blood samples (i.e. blood tubes or blood vials) or the number of different bloodtests (i.e. laboratory tests of blood samples).
23. Do the medical records indicate that cancer treatments were given during the 12 months following inclusion or until death?	❒ Yes – ***Go to question 24***<br>❒ No – ***Go to question 25***	
24. Which cancer treatments were given during the 12 months following inclusion or until death? How many times/days? *(More than 1 option possible)*	❒ Surgical operations, specify<br>.. .... times<br>.. .... times<br>.. .... times<br>.. .... times<br>.. .... times<br>❒ Intravenous chemotherapy ....days<br>❒ Oral chemotherapy ....days<br>❒ Radiation therapy ....days<br>❒ Immunotherapy ....days<br>❒ Targeted therapy (including hormone therapy), specify<br>.. ....days<br>.. ....days<br>.. ....days<br>.. ....days<br>.. ....days	
25. Were any of the following treatments given during the 12 months following inclusion or until death? *(More than 1 option possible)*	❒ Cardiopulmonary resuscitation .... times<br>❒ Artificial nutrition .... days<br>❒ Artificial hydration .... days<br>❒ Antibiotics .... days<br>❒ Mechanical ventilation .... days<br>❒ Blood transfusion .... times<br>❒ Continous deep sedation untill death<br>❒ Physician assisted death (e.g. euthanasia, physician assisted suicide)<br>❒ None of the above treatments	Artificial hydration related to intravenous chemotherapy only should <u>not</u> be taken into account.<br>Both oral and intravenous antibiotic therapies should be taken into account.<br>Only ventilation that is supported by a mechanical ventilation machine should be taken into account, oxygen therapy should <u>not</u> be taken into account.<br><br>Potential answer for continous deep sedation untill death or physician assisted death only in Belgium and the Netherlands.
**HOSPITALISATIONS**		
26. Do the medical records indicate that the patient was hospitalised (min. 1 night in hospital) during	❒ Yes – ***Go to question 27***<br>❒ No – ***Go to question 29***	Questions 26 to 28 refer to hospitalizations during the 12 months following inclusion or, in case the patients dies within 12 months after inclusion, untill the patient’s death.

the 12 months following inclusion or until death?		Stays in specialist palliative care units, offering specialist palliative care only, being part of a hospital or on the hospital campus, should not be counted as hospital admissions.<br><br>In order to retrieve information about hospitalizations, various types of notes will have to be searched, at least including: admission notes, discharge notes, etc.
27. How many times was the patient hospitalised (min. 1 night in hospital) during the 12 months following inclusion or until death?	❒ .... times<br>❒ Unclear	A hospitalization is defined as an overnight stay in hospital for at least one night.
28. How long did each period in hospital last?	Hospitalisation 1:<br>Days in ward:.......... Days in ICU:..........<br><br>Hospitalisation 2:<br>Days in ward:.......... Days in ICU:..........<br><br>Hospitalisation 3:<br>Days in ward:.......... Days in ICU:..........<br><br>Hospitalisation 4:<br>Days in ward:.......... Days in ICU:..........<br><br>Hospitalisation 5:<br>Days in ward:.......... Days in ICU:..........<br><br>Hospitalisation 6:<br>Days in ward:.......... Days in ICU:..........<br><br>Hospitalisation 7:<br>Days in ward:.......... Days in ICU:..........<br><br>Hospitalisation 8:<br>Days in ward:.......... Days in ICU:..........<br><br>Hospitalisation 9:<br>Days in ward:.......... Days in ICU:..........<br><br>Hospitalisation 10:<br>Days in ward:.......... Days in ICU:..........	The day of admission and the day of discharge have to be included to count the days in a ward or in an intensive care unit. The day on which a patient is transferred from a ward to an intensive care unit or vice versa, needs to be counted as a day in an intensive care unit and not as a day at the ward.
**SPECIALIST PALLIATIVE CARE**		
29. Do the medical records indicate that <u>specialist palliative caregivers</u>, either in hospital or elsewhere, were involved in the patient's care	❒ Yes – ***Go to question 30***<br>❒ No	Questions 29 and 30 refer to the involvement of <u>specialist</u> palliative caregivers in the patient's care, <u>either in hospital</u> (e.g. involvement of caregivers of the hospital multidisciplinary palliative support team, admission of the patient to the palliative care

during the 12 months following inclusion or until death?		unit of the hospital) or <u>elsewhere</u> (e.g. specialist palliative home care team, hospice, etc).<br><br>In order to find information about the involvement of specialist palliative caregivers, various types of notes will have to be searched, at least including: notes of in-hospital specialist palliative caregivers, discharge notes, etc.
30. On which date were specialist palliative caregivers for the first time involved in the patient's care?	❒ …../…../…..<br>❒ Information not available in records	Please indicate on which date involvement of the above mentioned specialist palliative caregivers was initiated.
**ADDITIONAL REMARKS**		
