# Supplementary material for: Healthcare use and healthcare costs for patients with advanced cancer; the international ACTION cluster-randomised trial on advance care planning
Source: Palliat Med. 2022 Dec 14;37(5):707–18. doi: 10.1177/02692163221142950 (PMC10227094; doi:10.1177/02692163221142950)
Supplement: sj-pdf-5-pmj-10.1177_02692163221142950 – Supplemental material for Healthcare use and healthcare costs for patients with advanced cancer; the international ACTION cluster-randomised trial on advance care planning [file sj-pdf-5-pmj-10.1177_02692163221142950.pdf]

Complete case analysis and exclude deaths within 12 months of inclusion in study

		Univariable			Multivariable	
**Characteristic**	**exp(Beta)**	**95% CI**	**p-value**	**exp(Beta)**	**95% CI**	**p-value**
**Study group**						
Control group	Ref					
Intervention group	1.74	0.51, 6.01	0.4	0.91	0.76, 1.10	0.3
**Age_category**						
46-65 yrs	Ref			0.85	0.50, 1.43	0.5
>65 yrs	0.82	0.27, 2.54	0.7	0.85	0.50, 1.43	0.5
18-45 yrs						
**Sex**						
Male	Ref					
Female	1.17	0.38, 3.60	0.8	0.87	0.72, 1.04	0.12
**Country**						
Belgium				1.56	1.19, 2.05	0.001
Denmark	1.76	0.37, 8.39	0.5	2.11	1.56, 2.85	<0.001
United Kingdom	0.34	0.10, 1.13	0.078	0.75	0.58, 0.96	0.023
The Netherlands	Ref					
Slovenia				0.21	0.14, 0.30	<0.001
Italy				0.85	0.63, 1.15	0.3
**Religious**						
Yes	Ref					
No	1.23	0.33, 4.54	0.8	0.82	0.68, 0.99	0.043
Prefer not to specify	1.85	0.43, 7.84	0.4	0.70	0.54, 0.92	0.009

Characteristic	Univariable exp(Beta)	Univariable 95% CI	Univariable p-value	Multivariable exp(Beta)	Multivariable 95% CI	Multivariable p-value
**Cancer_type**						
Small cell - lung cancer	Ref					
Non-small cell lung cancer	0.38	0.05, 3.04	0.4	1.25	0.92, 1.70	0.15
Colon cancer	0.12	0.01, 1.18	0.069	1.33	0.39, 4.50	0.6
Rectal cancer	0.24	0.01, 4.09	0.3	1.51	0.45, 5.08	0.5
**Current_stage**						
Stage III, lung cancer	Ref					
Stage IV, lung cancer	2.64	0.70, 9.91	0.15	1.02	0.77, 1.34	>0.9
Colorectal cancer stage IV	1.09	0.23, 5.16	>0.9	0.73	0.22, 2.44	0.6
Colorectal cancer - metachronous metastases	0.01	0.00, 0.12	<0.001	0.96	0.28, 3.29	>0.9
**WHO performance status**						
0 Fully active	Ref					
1 No heavy physical work	0.56	0.18, 1.68	0.3	0.85	0.70, 1.04	0.11
2 Up for more than half the day	1.84	0.19, 17.6	0.6	0.77	0.57, 1.03	0.078
3 In bed/sitting more than half the day				0.37	0.18, 0.76	0.007
