# Supplementary material for: Healthcare use and healthcare costs for patients with advanced cancer; the international ACTION cluster-randomised trial on advance care planning
Source: Palliat Med. 2022 Dec 14;37(5):707–18. doi: 10.1177/02692163221142950 (PMC10227094; doi:10.1177/02692163221142950)
Supplement: sj-pdf-6-pmj-10.1177_02692163221142950 – Supplemental material for Healthcare use and healthcare costs for patients with advanced cancer; the international ACTION cluster-randomised trial on advance care planning [file sj-pdf-6-pmj-10.1177_02692163221142950.pdf]

Appendix 4 Costs of ACP conversations and total costs of use of care in the intervention group by country

Country	Costs of ACP MIN [1] Mean [IQR]	Costs of ACP MAX [2] Mean [IQR]	Total costs including costs of ACP MIN [3] Mean [IQR]	Total costs including costs of ACP MAX [4] Mean [IQR]
Belgium	34<br>[0, 54]	74<br>[0, 118]	54930<br>[20496, 81597]	54970<br>[20531, 81608]
Denmark	34<br>[24, 42]	66<br>[48, 82]	61211<br>[31771, 74966]	61244<br>[31807, 74992]
Italy	34<br>[26, 42]	84<br>[64, 104]	41882<br>[14239, 61317]	41932<br>[14304, 61356]
The Netherlands	67<br>[41, 90]	200<br>[122, 270]	25428<br>[7210, 35763]	25562<br>[7313, 35844]
Slovenia	35<br>[24, 43]	68<br>[47, 83]	6102<br>[39, 9361]	6135<br>[72, 9394]
UK	73<br>[52, 90]	171<br>[122, 213]	17010<br>[4248, 20432]	17109<br>[4324, 20546]

[1] Costs of ACP conversations based on wages of nurses
[2] Costs of ACP conversations based on wages of medical specialists
[3] Total costs of care for patient in intervention group including minimal costs of ACP conversations
[4] Total costs of care for patient in intervention group including maximal costs of ACP conversations
